# Lack of cortistatin or somatostatin differentially influences DMBA-induced mammary gland tumorigenesis in mice in an obesity-dependent mode

**DOI:** 10.1186/s13058-016-0689-1

**Published:** 2016-03-08

**Authors:** Raúl M. Luque, Alicia Villa-Osaba, Fernando L-López, Ana I. Pozo-Salas, Rafael Sánchez-Sánchez, Rosa Ortega-Salas, Luis de Lecea, Marina Álvarez-Benito, José López-Miranda, Manuel D. Gahete, Justo P. Castaño

**Affiliations:** Department of Cell Biology, Physiology, and Immunology, University of Córdoba, Reina Sofía University Hospital, Maimonides Biomedical Research Institute of Córdoba (IMIBIC), Córdoba, Spain; CIBER Fisiopatología de la Obesidad y Nutrición (CIBERobn), Córdoba, Spain; Campus de Excelencia Internacional Agroalimentario, Córdoba, Spain; University of Córdoba, Reina Sofía University Hospital, Maimonides Biomedical Research Institute of Córdoba (IMIBIC), Córdoba, Spain; Anatomical Pathology Service, Reina Sofia University Hospital, Maimonides Biomedical Research Institute of Córdoba (IMIBIC), Córdoba, Spain; Department of Psychiatry and Behavioral Sciences, Stanford University School of Medicine, Palo Alto, CA USA; Mammary Gland Unit, Reina Sofía University Hospital, University of Córdoba, Maimonides Biomedical Research Institute of Córdoba (IMIBIC), Córdoba, Spain; Lipids and Atherosclerosis Unit, Department of Medicine, Reina Sofía University Hospital, University of Córdoba, Maimonides Biomedical Research Institute of Córdoba (IMIBIC), Córdoba, Spain

**Keywords:** Cortistatin, Somatostatin, Mammary gland, Tumorigenesis, Obesity, Mouse models

## Abstract

**Background:**

Somatostatin (SST) and cortistatin (CORT), two structurally and functionally related peptides, share a family of widespread receptors (sst1-5) to exert apparently similar biological actions, including endocrine/metabolic regulation and suppression of tumor cell proliferation. However, despite their therapeutic potential, attempts to apply SST-analogs to treat breast cancer have yielded unsatisfactory results. Actually, the specific roles of SST and CORT in mammary gland tumorigenesis (MGT), particularly in relation to metabolic dysregulation (i.e. obesity), remain unknown.

**Methods:**

The role of endogenous SST and CORT in carcinogen-induced MGT was investigated under normal (lean) and obesity conditions. To that end, SST- and CORT-knockout (KO) mice and their respective littermate-controls, fed low-fat (LF) or high-fat (HF) diets, were treated with 7,12-dimethyl-benza-anthracene (DMBA) once a week (wk) for 3 wk, and MGT was monitored for 25 wk. Additionally, we examined the effect of SST or CORT removal in the development of the mammary gland.

**Results:**

Lack of SST did not alter DMBA-induced MGT incidence under lean conditions; conversely, lack of endogenous CORT severely aggravated DMBA-induced MGT in LF-fed mice. These differences were not attributable to altered mammary gland development. HF-diet modestly increased the sensitivity to DMBA-induced carcinogenesis in control mice, whereas, as observed in LF-fed CORT-KO, HF-fed CORT-KO mice exhibited aggravated tumor incidence, discarding a major influence of obesity on these CORT actions. In marked contrast, HF-fed SST-KO mice exhibited much higher tumor incidence than LF-fed SST-KO mice, which could be associated with higher mammary complexity.

**Conclusions:**

Endogenous SST and CORT distinctly impact on DMBA-induced MGT, in a manner that is strongly dependent on the metabolic/endocrine milieu (lean vs. obese status). Importantly, CORT, rather than SST, could represent a major inhibitor of MGT under normal/lean-conditions, whereas both neuropeptides would similarly influence MGT under obesity conditions. The mechanisms mediating these different effects likely involve mammary development and hormones, but the precise underlying factors are still to be fully elucidated. However, our findings comprise suggestive evidence that CORT-like molecules, rather than classic SST-analogs, may help to identify novel tools for the medical treatment of breast cancer.

**Electronic supplementary material:**

The online version of this article (doi:10.1186/s13058-016-0689-1) contains supplementary material, which is available to authorized users.

## Background

Breast cancer is the most prevalent type of cancer in the female population [1, 2]. With a hereditary component of only 5 %, this complex and heterogeneous disease has distinct clinical and histological forms with a diverse clinical prognosis [3]. Currently, efforts to develop medical tools to combat this disease are primarily focused on identifying novel therapeutic targets. Somatostatin (SST) and cortistatin (CORT), two highly related pleiotropic neuropeptide hormones [4], have been shown to inhibit proliferation and invasion of breast cancer and other cell types [5], suggesting their potential value to treat various endocrine-related tumors. Unfortunately, clinical attempts to treat breast cancer with available SST analogs, such as octreotide or lanreotide, successfully used in the treatment of pituitary and neuroendocrine tumors [6, 7], have generated inconsistent, largely unsatisfactory clinical results in breast cancer patients [8–10].

SST was originally discovered by its ability to inhibit the secretion of growth hormone (GH) or somatotropin (thus its name) [11]; but a number biological functions were subsequently identified for SST, including inhibition of secretion from numerous endocrine and exocrine cells, and regulation of cell proliferation and differentiation [12]. CORT [13] is highly similar to SST in structure, sequence and pharmacological properties, and share a number of functional capacities with SST [14, 15]. Notwithstanding, SST and CORT are not merely two endocrine, redundant siblings, in that CORT and SST exhibit different tissue distribution patterns and are able to exert several distinct, even opposite, actions [4, 16]. In particular, CORT exerts unique immunomodulatory and anti-inflammatory effects, which are not shared by SST, and could be related to expression of CORT in immune cells [17–19]. Both SST and CORT exert their actions by binding with comparable affinity to the so-called SST receptors (sst1-5). However, it has been demonstrated that CORT can also uniquely interact with additional, non-SST receptors, such as the ghrelin receptor (GHSR1a) or the Mas-related gene *X*2 (MrgX2) receptor [20, 21]. Interestingly, several studies using mice models have shown that SST and CORT are key factors that distinctly contribute to maintaining the correct regulation of whole body homeostasis in response to metabolic challenges, such as obesity, by acting on particular target tissues [22–24].

The SST/CORT system seems therefore to stand at the regulatory crossroads between tumor development and deregulated metabolic conditions, where it could play a singular role in the pathological interaction between obesity and breast cancer, an endocrine-related pathological condition profoundly influenced by the metabolic status [25, 26]. However, the controversial, disappointing results observed in the scarce clinical trials that have tested SST analogs in the treatment of patients with breast cancer suggest that more studies are necessary to fully elucidate the exact role of SST and CORT in the development and/or progression of breast cancer, especially in the context of obesity. Accordingly, the aim of the present study was to clarify the precise role that SST and CORT might play in the development and/or progression of mammary carcinogenesis under normal feeding and obesity conditions. To this end, we have examined, for the first time, the impact of treatment with 7, 12 dimethylbenz[α]anthracene (DMBA), an immunosuppressor and a powerful organ-specific laboratory carcinogen, on mammary gland (MG) hyperplasia and tumor formation/progression in the presence or absence of endogenous SST or CORT, using SST knockout (KO) and CORT KO mice under normal and diet-induced obese conditions.

## Methods

### Animal generation and maintenance

All experimental procedures were approved by the Animal Care and Use Committees of the University of Cordoba. All mice were bred in-house and maintained under standard conditions of light (12-h light, 12-h dark cycle; lights on at 07:00 h) and temperature (22–24 °C), with free access to tap water and food. C57Bl6/J SST-KO (kindly provided by Dr. Ute Hochgeschwender) and CORT-KO mice were crossbred, at the same time, during 10 generations with same littermate FVB/N mice (Harlan Laboratories, Inc. Indianapolis, IN, USA) in order to generate SST-KO and CORT-KO mice in a pure FVB/N strain, which has been shown to be more susceptible to DMBA-induced MG tumorigenesis [27, 28] than the C57Bl6/J [29, 30]. FBV/N CORT-KO and SST-KO and their corresponding littermate wild-type (WT) controls (cort+/+ or sst+/+, respectively) generated from heterozygous breeding pairs were used in this study. All WT control mice (cort+/+ and sst+/+) were pooled together and considered as the control (WT) group. For all experimental groups and prior to killing, all mice were trained and handled at least one week to acclimate them to personnel and handling methods. All female mice under the random cycling condition were killed by decapitation without anesthesia.

### Induction of mammary gland (MG) tumors by DMBA treatment

To test the effect of endogenous SST and CORT ablation on carcinogen-induced MG tumorigenesis (MGT) and its interaction with the metabolic status, 8-week-old female WT, CORT-KO and SST-KO mice (*n* = 23–43 per group) were fed a low-fat (LF) diet (Research Diets, Gentofte, Denmark; D12450B; 10 % Kcal fat, 70 % Kcal carbohydrates, 20 % Kcal proteins) or a micronutrient-matched high-fat (HF) diet (Research Diets; D12492; 60 % Kcal fat, 20 % Kcal carbohydrates, 20 % Kcal proteins) for 12 weeks and then, treated with DMBA (Sigma-Aldrich, Madrid, Spain) dissolved in olive oil (0.5 mg/10 g body weight (BW), by oral gavage) once a week for 3 consecutive weeks [31, 32]. Subsequently, the development and progression of MG tumors was monitored for an additional 25 weeks, wherein the LF and HF feeding regimens were maintained until the end of the experiment. Mice were sacrificed at 47 weeks of age (unless mice developed excessive tumor burden (>1 cm^3^, as set by the Institutional Animal Care and Use Committee (IACUC) standard)), when tumors and MGs were dissected and trunk blood collected for further analysis. Body weights were recorded twice a week.

### Determination of whole body composition

Whole body composition (fat, lean and extracellular water content) was assessed in live DMBA-treated mice using a Body Composition Analyzer E26-240-RMT (EchoMRI LLC, Houston, TX, USA) the day before killing the mice (47 weeks of age).

### MG development studies

An additional group of WT, CORT-KO and SST-KO female mice (*n* = 10–30 per group) were killed at 8 weeks of age to investigate the effect of genotype in MG development. Finally, a third group of 8-week-old WT, cort−/− and sst−/− female mice (*n* = 4–12 per group) were fed a LF or HF diet for 12 weeks and killed at 20 weeks of age to investigate the effect of the genotype in MG development in combination with a HF diet.

### Whole-mount MG analysis

As recently reported [33], the inguinal MGs were excised from mice on sacrifice for whole-mount analysis, using sharp scissors, starting from the proximal area close to the nipple towards the distal end of the gland close to the spine of the animal, collecting all the adipose tissue delimiting the inguinal mammary area. Subsequently, the glands were spread onto a glass slide, fixed in PenFix (Richard-Allan Scientific, Kalamazoo, MI, USA) for 24 hours, defatted in acetone and rehydrated in graded ethanol, ending with distilled water. The whole mounts were then stained with carmine (0.2 % carmine, 0.5 % aluminum potassium sulfate) (Merck, Billerica, MA, USA), washed in running water and dehydrated in ascending graded ethanol solutions. Stained whole mounts were stored and photographed in Histolemon (Carlo Erba, Sabadell, Spain). Photographs were used to assess the number of terminal end buds (TEBs) and the extent of ductal branching, by counting the number of intersecting branches along a line drawn between the leading edge of the ducts and the lymph node, as reported recently [33]. The number of branches per unit length was used as an indicator of gland complexity. Two blinded observers determined the presence of non-palpable tumors and hyperplastic lesions on the whole mounts obtained from DMBA-treated mice.

### MG tumor histopathology

A subset of the tumors from DMBA-treated mice were dissected, fixed in 10 % formalin, paraffin-embedded and sectioned in 7-μm sections for hematoxylin-eosin staining or immunohistochemical analysis by the Laboratory of Histology at UCAIB (IMIBIC). Human epidermal growth factor receptor (HER2) (R&D Systems, Minneapolis, USA, catalog number AF5176), estrogen receptor (ER) (Millipore, Billerica, MA, USA, catalog number 06-935), progesterone receptor (PR) (Thermo Scientific, Waltham, MA, USA, catalog number MA1-411) and Ki67 (Abcam, Cambridge, UK, catalog number ab15580) presence was evaluated in this subset of tumors using commercial antibodies. The number of mitotic cells (per each 10 high power fields), inflammation grade (presence of inflammatory cells in the tumor) and *de novo* vascularization (presence of small blood vessels in the tumor) were also determined following standard protocols. Two independent pathologists performed the histopathological analysis of the tumors following a blinded protocol.

### Assessment of circulating hormones

Trunk blood was collected from WT, CORT-KO and SST-KO mice after killing (47 weeks of age) and immediately mixed with MiniProtease inhibitor (Roche, Barcelona, Spain) for serum samples or with ethylenediaminetetraacetic acid (EDTA) for plasma samples, placed on ice, centrifuged and stored at −20 °C until assessment of hormones. Commercial ELISA kits were used to assess circulating IGF-I, corticosterone (Immunodiagnostic Systems, Gaithersburg, MD, USA), leptin, GH, insulin (Millipore) and prolactin (CalBiotech, Spring Valley, CA, USA) following the manufacturer’s instructions.

### Data and statistical analysis

Tumor latency was calculated as the time between the last DMBA dose and the date the tumor was first detected by palpation of the MGs. Tumor multiplicity indicates the average number of palpable tumors found per mouse within the group at the termination of the study (including those animals that did not develop tumors). Tumor burden indicates the average number of palpable tumors found per tumor-bearing mouse, within the group, at the termination of the study. Death counts during the observation period included mice that were euthanized due to excessive tumor burden (>1 cm^3^, set by the IACUC standard) or those found moribund due to unknown causes. Mice that formed tumors prior to death were included to calculate tumor latency, while other endpoints were assessed in mice that completed the study (60–78 % of initial animals). Two-way analysis of variance (ANOVA) and Kruskal–Wallis analyses were used to evaluate differences between diet (LF and HF) or genotype (WT, CORT-KO and SST-KO), followed by Fisher’s, Bonferroni or Mann–Whitney post-hoc test. Log-rank (Mantel–Cox) analysis and Fisher’s exact test were used to evaluate the differences in tumor incidence between WT, CORT-KO and SST-KO mice and between diets. All values are expressed as percentage or mean ± standard error of the mean (SEM). *p* <0.05 was considered significant. All statistical analyses were performed using GraphPad Prism 6.0 software (GraphPad Software Inc., La Jolla, CA, USA).

## Results

### Confirmation of diet-induced obese phenotype

**Fig. 1:**
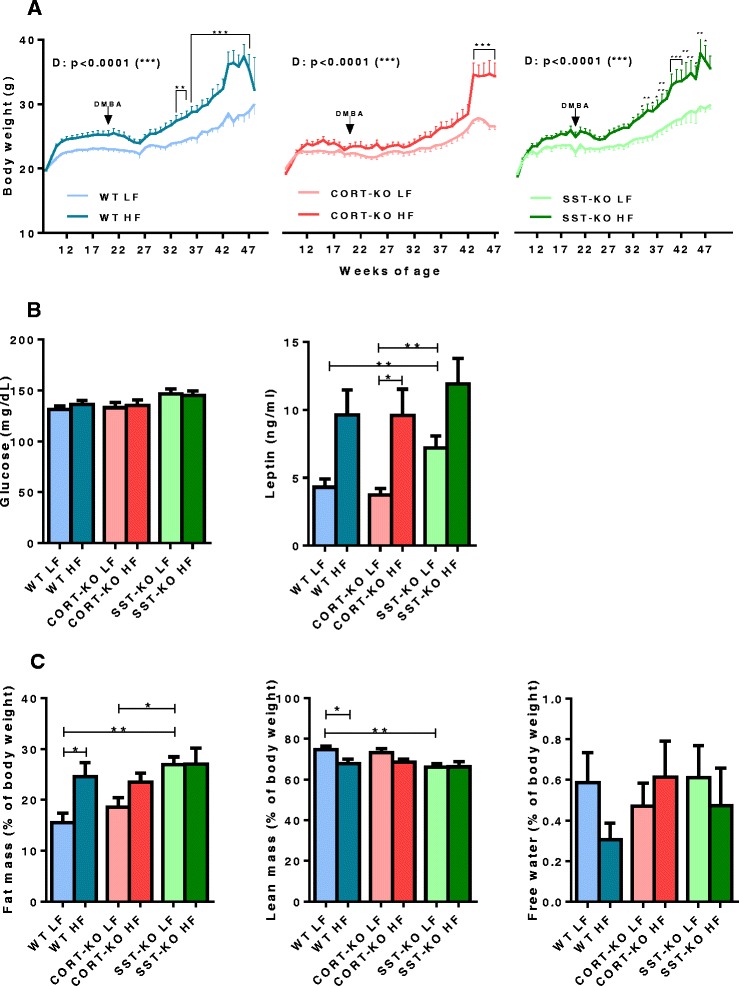
Diet-induced obese phenotype in wild-type (*WT*), cortistatin (*CORT*)-knockout (*KO*) and somatostatin (*SST*)-KO mice. **a** Growth curves (represented as body weight over time of high-fat (*HF*) diet feeding, starting at 8 week of age). *D* indicates effect of HF diet analyzed by two-way analysis of variance. **b** Glucose (mg/dL) and leptin levels (ng/mL) at the day of sacrifice (23–43/group). **c** Fat mass, lean mass and free water percentages (close to the day of sacrifice; *n* = 10–16/group). Values are mean ± standard error of the mean: *p <0.05, **p <0.01, ***p <0.001 for significant differences between groups analyzed by the Fisher, Bonferroni or Mann–Whitney post-hoc test

To test the effect of endogenous CORT and SST ablation on carcinogen-induced MGT and the interaction with metabolic status, 8-week-old female WT, CORT-KO and SST-KO mice were fed a LF or HF diet for 12 weeks, and were then treated with DMBA. To confirm the obese status induced by the HF diet, all mice were weighed twice a week and the resultant growth curves, represented in Fig. 1a, indicated that mice fed a HF diet were heavier than those fed a LF diet in all groups at the time of the sacrifice (*p* <0.001 in all cases). As illustrated in Fig. 1b, basal glucose levels were not significantly altered by a HF diet in any of the experimental groups. In contrast, plasma leptin levels were elevated in animals on the HF compared to those on the LF diet, although the increase was only statistically significant in CORT-KO mice. An elevation of leptin levels was also observed in LF-fed SST-KO mice compared to LF-fed control and CORT-KO mice.

Percentages of fat mass, lean mass and water composition were monitored in a subset of these animals (*n* = 10–16/group) the day before sacrifice (Fig. 1c). There was a significant increase in percentage fat mass (*p* = 0.012) and decrease in percentage lean mass (*p* = 0.038) in HF-fed WT compared to LF-fed mice, whereas there were no significant changes observed in SST-mice and CORT-mice. Overall, these data suggest the obese phenotype was accompanied by clear changes in lean and fat mass percentage in WT mice, but not in SST- and CORT-mice.

### Effect of the lack of SST or CORT in DMBA-induced MG tumor development in lean and obese mice

**Fig. 2:**
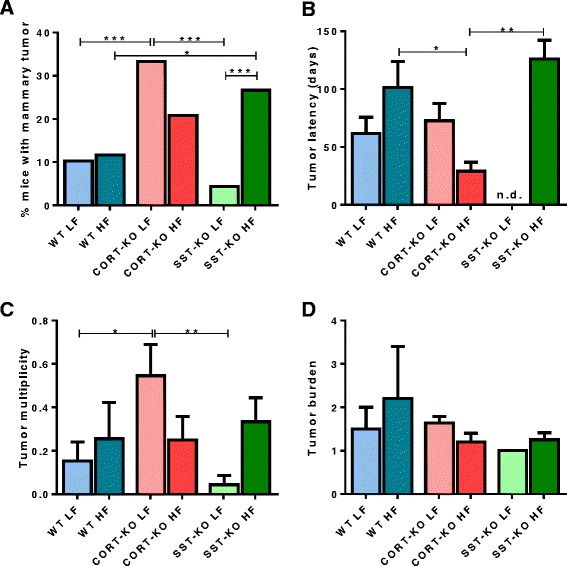
Mammary gland tumor formation induced by 7, 12 dimethylbenz[α]anthracene (DMBA) in wild-type (*WT*), cortistatin (*CORT*)-knockout (*KO*) and somatostatin (*SST*)-KO mice on a low fat (*LF*)/high fat (*HF*) diet. **a** Tumor incidence (represented as percentage of mice with tumors), **b** tumor latency in days, **c** tumor multiplicity, and **d** tumor burden (*n* = 23–43/group). Values represent percentages (for tumor incidence) or mean ± standard error of the mean: *p <0.05, **p <0.01, ***p <0.001 for significant differences between groups analyzed by the Fisher or Mann–Whitney post-hoc test. *n.d.* not detected

WT, CORT-KO, and SST-KO mice fed on a LF or a HF diet were monitored for 25 weeks after DMBA administration, until sacrifice. Alternatively, mice were killed if the tumor reached a size >1 cm^3^ (according to the IACUC standards). There was a 10.25 % incidence of DMBA-induced tumors in LF-fed WT mice (Fig. 2a), with a tumor latency of 61.5 days (Fig. 2b). Interestingly, there were no significant differences in WT mice fed a HF diet compared to lean (LF diet) mice in tumor incidence (11.62 % vs 10.25 %, respectively; Fig. 2a), tumor latency (Fig. 2b), multiplicity (Fig. 2c), or burden (Fig. 2d). However, on analysis of the MG whole mounts (Table 1), although the number of tumors was similar, the presence of ductal mammary hyperplasia was significantly increased by HF feeding, with a consequent reduction in the percentage of normal MGs in obese mice.

**Table 1:** DMBA-induced mammary gland tumor formation in WT, CORT-KO and SST-KO mice by whole-mount analysis

	WT		CORT-KO		SST-KO	
	LF (*n* = 39)	HF (*n* = 43)	LF (*n* = 33)	HF (*n* = 24)	LF (*n* = 23)	HF (*n* = 30)
Whole-mount analysis						
Tumor	3 %	5 %	14 %^##^	13 %	4 %^$^	3 %^$^
Hyperplasia	16 %	35 %^**^	10 %	25 %^**^	13 %	38 %^***^
Normal	81 %	60 %^**^	76 %	63 %	83 %	59 %^**^
Percentage of mice that were not included in the study^a^	35 %	24.5 %	21.4 %	40 %	30.3 %	23 %

**p <0.01, ***p <0.001, for mice fed a low fat (*LF*) diet vs high fat (*HF*) diet (within the same genotype); ^##^ *p* <0.01 in wild-type (*WT*) vs knockout (*KO*) mice (within the same diet group); ^$^ *p* <0.05) in cortistatin (*CORT*)-KO vs somatostatin (*SST*)-KO (within the same diet group). ^a^Mice that died during the 3 weeks of 7, 12 dimethylbenz[α]anthracene (DMBA) administration or during the weeks immediately subsequent, due to the toxicity of this compound and/or the invasiveness of the method used to deliver DMBA, were not included in the study

On the other hand, tumor incidence increased drastically (i.e., tripled) in lean mice lacking CORT (LF-fed CORT-KO) as compared to lean WT mice (Fig. 2a). Specifically, 10.25 % of WT mice developed a DMBA-induced mammary tumor, whereas 33.33 % of CORT-KO mice had tumors. In addition, tumor multiplicity was higher in CORT-KO LF mice compared to lean controls, without similar changes in tumor latency and tumor burden (Fig. 2b and d). Consistently, MG whole mounts confirmed an increase in mammary tumors in CORT-KO LF mice compared to their WT LF counterparts (Table 1). In HF-fed CORT-KO mice, DMBA-induced tumor incidence was numerically higher than in HF-fed controls, although this difference was not significant (*p* = 0.126, Fisher’s test). Conversely, tumor latency in obese CORT-KO (Fig. 2b) was significantly lower compared to obese WT mice (*p* = 0.028), whereas tumor multiplicity (Fig. 2c), tumor burden (Fig. 2d) and whole-mount variables were not significantly different (Table 1). Interestingly, HF-fed CORT-KO mice had levels of tumor incidence, latency, multiplicity, and burden that were numerically, but not significantly lower than those observed in lean CORT-KO. In fact, whole-mount analysis indicated that HF-feeding significantly increased ductal mammary hyperplasia in CORT-KO mice, but did not influence tumor formation (Table 1).

In striking contrast with that observed for CORT, lack of endogenous SST did not influence DMBA-induced tumorigenesis in lean mice compared with WT controls, as only 4.34 % of LF-fed SST-KO mice developed mammary tumors, a similarly low percentage to that observed in LF-fed WT mice and substantially lower than in LF-fed CORT-KO animals (Fig. 2a). Similarly, no significant differences in tumor latency, multiplicity, or burden were observed between WT and SST-KO LF-fed groups. Interestingly, under obese conditions, SST-KO exhibited a remarkable increase in tumor incidence compared to lean SST-KO (26.66 % vs 4.34 %; *p* <0.001) and to their HF-fed WT counterparts. In addition, obese SST-KO mice had increased tumor multiplicity (*p* = 0.053, Fig. 2c) and ductal mammary hyperplasia (Table 1) compared to lean, LF-fed SST-KO mice, and a concomitantly lower percentage of normal MGs. Of note, on comparison of both KO mice models there was a strikingly higher proportion of MG tumors (Fig. 2a), and higher tumor multiplicity (Fig. 2c), in LF-fed CORT-KO compared to LF-fed SST-KO mice. In contrast, under obesity conditions, CORT-KO and SST-KO had a similar percentage of mammary tumors, although latency in tumor development was lower in CORT-KO than in SST-KO mice (Fig. 2b). Consistently, the percentage of hyperplasia was comparable in CORT-KO and SST-KO mice, whereas CORT-KO mice had a higher proportion of MG tumors compared to SST-KO mice (Table 1).

**Fig. 3:**
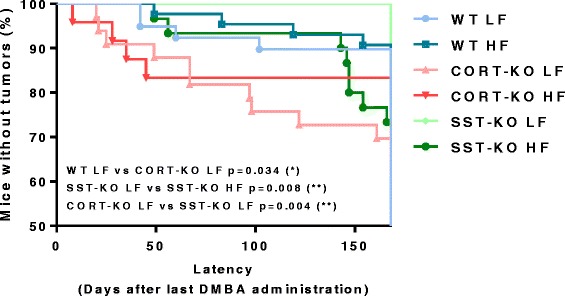
Incidence curve for 7, 12 dimethylbenz[α]anthracene (*DMBA*)-induced mammary gland tumor in wild-type (*WT*), cortistatin (*CORT*)-knockout (*KO*) and somatostatin (*SST*)-KO mice on a low fat (*LF*)/high fat (*HF*) diet. Percentage of mice (*n* = 23–43/group) without tumor after DMBA administration over time for each experimental group, indicating the moment when tumors were first detected. Differences identified by the log-rank (Mantel–Cox) test are shown

These results were further supported by the analysis of the tumor incidence curves, where the percentage of mice without tumors was represented over time (Fig. 3). Specifically, on log-rank (Mantel-Cox) analyses there were significant differences between LF-fed WT and LF-fed CORT-KO mice (*p* = 0.034), between CORT-KO and SST-KO under LF conditions (*p* = 0.004) and between SST-KO mice fed a LF or a HF diet (*p* = 0.008).

Histopathological analysis of a subset of these tumors showed all samples to be adenocarcinomas, squamous carcinomas or undifferentiated solid tumors (Additional file 1: Figure S1), consistent with previous reports showing that DMBA predominantly induces adenocarcinoma and adenosquamous carcinoma in several mouse strains [28, 34, 35]. There was no apparent effect of diet or genotype on tumor histological type (Additional file 1: Figure S1) or on HER2/ER/PR status (Table 2), as 90 %, 95 % and 56 % of tumors were HER2-negative, ER-positive and PR-positive, respectively. Similarly, analyses of proliferation rate, inflammation and *de novo* angiogenesis in a subset of the tumoral pieces (Table 2 and Additional file 2: Figure S2) were not significantly different among the experimental groups.

**Table 2:** HER2, ER and PR status and malignancy features of DMBA-induced mammary gland tumors in WT, CORT-KO and SST-KO mice

Group	Histological type	HER2^a^	ER	PR	Ki 67	Mitosis^b^	Inflammation^c^	Angiogenesis^c^
WT LF	Undifferentiated carcinoma	1+ Negative	Positive	Positive	-	10	1/3	1/3
	Undifferentiated carcinoma	1+ Negative	Positive	Negative	-	12	1/3	2/3
	Well-differentiated squamous carcinoma	1+ Negative	Positive	-	-	9	2/3	1/3
WT HF	Undifferentiated carcinoma	0 Negative	Positive	Negative	-	11	0/3	1/3
	Moderately differentiated adenocarcinoma	1+ Negative	Positive	Negative	40 %	6	1/3	1/3
CORT-KO LF	Undifferentiated carcinoma	0 Negative	Positive	Positive	65 %	12	1/3	1/3
	Undifferentiated carcinoma	0 Negative	Positive	Positive	70 %	19	1/3	2/3
	Undifferentiated carcinoma	0 Negative	Positive	-	65 %	5	1/3	1/3
	Undifferentiated carcinoma	1+ Negative	Positive	Negative	-	5	1/3	2/3
	Undifferentiated carcinoma	1+ Negative	Negative	-	-	5	2/3	1/3
	Undifferentiated carcinoma	1+ Negative	Positive	Negative	-	8	0/3	1/3
	Undifferentiated carcinoma	0 Negative	Positive	Positive	70 %	7	1/3	1/3
	Well-differentiated squamous carcinoma	1+ Negative	Positive	Positive	70 %	9	1/3	1/3
	Well-differentiated squamous carcinoma	1+ Negative	Positive	Positive	90 %	10	1/3	1/3
	Well-differentiated squamous carcinoma	1+ Negative	-	Positive	70 %	8	1/3	1/3
CORT-KO HF	Undifferentiated carcinoma	0 Negative	Positive	Negative	75 %	12	2/3	1/3
	Undifferentiated carcinoma	0 Negative	Positive	Positive	-	6	1/3	1/3
	Well-differentiated squamous carcinoma	2+ Positive	Positive	Negative	70 %	13	2/3	1/3
	Moderately differentiated squamous carcinoma	1+ Negative	Positive	Positive	-	2	3/3	2/3
SST-KO HF	Undifferentiated carcinoma	1+ Negative	Positive	-	50 %	7	1/3	1/3
	Undifferentiated carcinoma	1+ Negative	Positive	Negative	80 %	19	1/3	2/3
	Well-differentiated squamous carcinoma	2+ Positive	Positive	Positive	75 %	10	1/3	1/3

^a^Presence of human epidermal growth factor receptor-2 (*HER2*) was evaluated as negative (0: negative staining or, 1+: >10 % of cells with incomplete or undetectable staining) or positive (2+: >10 % of cells with complete staining). ^b^Number of mitotic cells per each 10 high power fields. ^c^Inflammation and *de novo* angiogenesis were valued as 0/3 (no inflammation), 1/3 (scarce), 2/3 (moderate) or 3/3 (abundant). *ER* estrogen receptor, *PR* progesterone receptor, *WT* wild-type, *HF* high fat, *LF* low fat, *CORT-KO* cortistatin knockout, *SST-KO* somatostatin knockout

### MG complexity

**Fig. 4:**
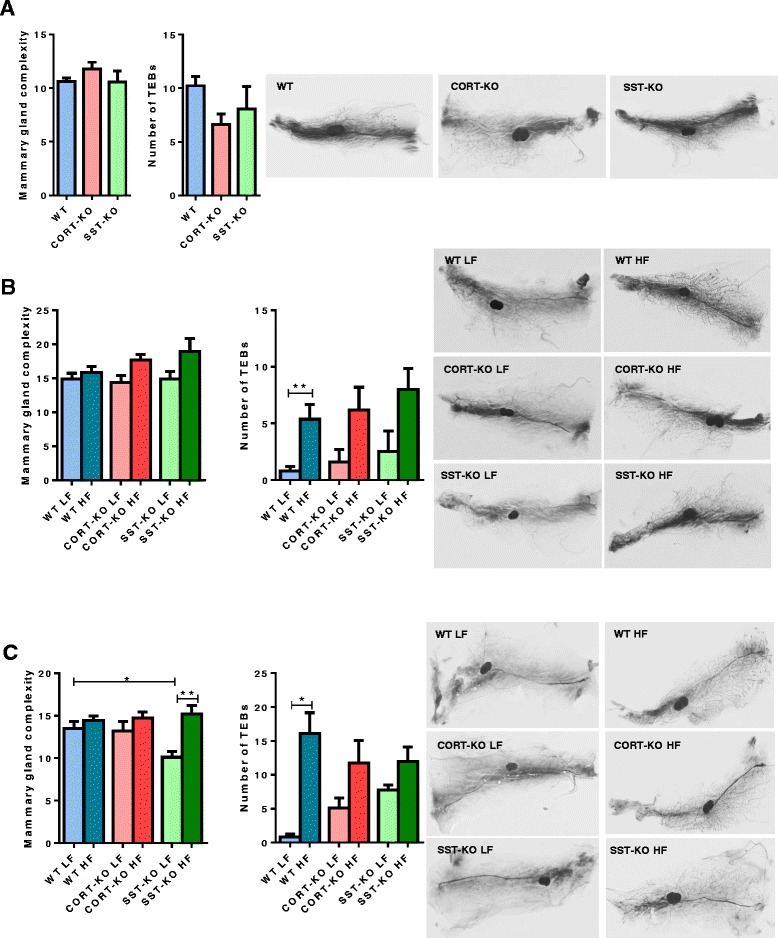
Impact of low fat (*LF*)/high fat (*HF*) diets on mammary gland (MG) development in wild-type (*WT*), cortistatin (*CORT*)-knockout (*KO*) and somatostatin (*SST*)-KO mice. MG complexity (represented as the ductal branching by counting number of intersecting branches along a line between the leading edge of the ducts and the lymph node) and terminal end buds (TEBs) were determined in: **a** young virgin (8-week-old) WT, CORT-KO and SST-KO mice (*n* = 10–30/group); **b** adult WT, CORT-KO and SST-KO mice fed a LF or a HF diet for 12 weeks (*n* = 4–12/group); and **c** adult WT, CORT-KO and SST-KO mice (4–14/group) fed a LF or HF diet for 12 weeks and treated with 7, 12 dimethylbenz[α]anthracene (DMBA) at 20 weeks of age. Values represent mean ± standard error of the mean: *p <0.05, **p <0.01 for differences between groups analyzed by the Bonferroni or Mann–Whitney test. *Right* representative images of each experimental group by whole mount

To further explore the putative causes underlying the differences observed in DMBA-induced tumor incidence, we investigated the effect of diet-induced obesity and lack of CORT or SST in MG development, by analyzing MG complexity and the number of TEBs on whole mounts. MG complexity and the number of TEBs were not statistically different among 8-week old WT, CORT-KO, or SST-KO female mice (n = 10–30 mice/group; Fig. 4a). As expected, 12 weeks on the HF diet promoted an increase in the number of TEBs in mice on the HF compared to the LF diet, although it was only statistically significant in the WT group (*n* = 4–12 mice/group; Fig. 4b). Consistently, after the DMBA treatment, on whole-mount analyses obese mice had more TEBs, which was significantly evident in the WT mice (Fig. 4c). In addition, at the end of the study, lean SST-KO had decreased MG complexity compared to lean WT and HF-fed SST-KO mice.

### Circulating hormone levels

**Fig. 5:**
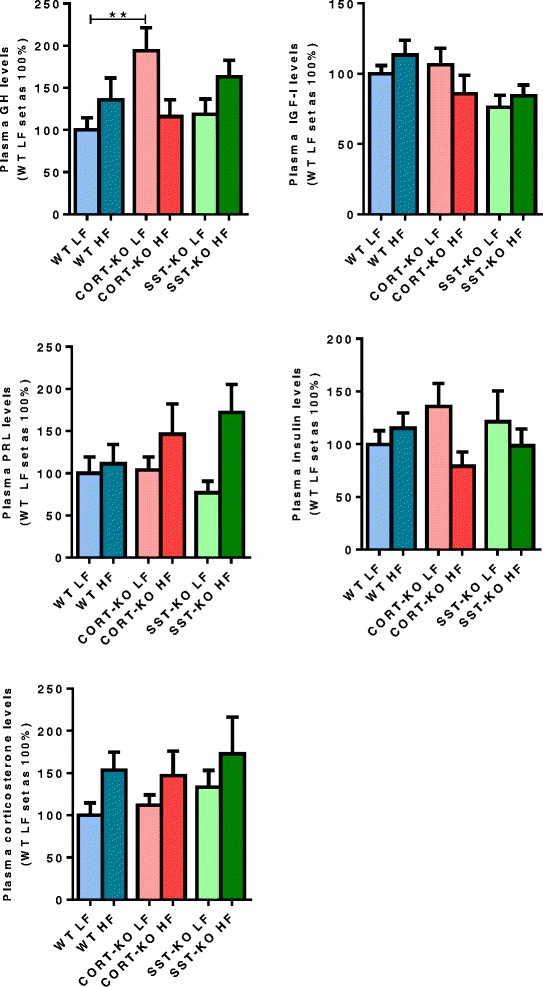
Circulating hormone levels in wild-type (*WT*), cortistatin (*CORT*)-knockout (*KO*) and somatostatin (*SST*)-KO mice on low fat (*LF*) and high fat (*HF*) diets. Values represent mean ± standard error of the mean of the percentage of growth hormone (*GH*), insulin-like growth factor-1 *(IGF-I*), prolactin (*PRL*), insulin and corticosterone plasma levels of each experimental group (*n* = 22–40/group; setting the WT LF group as 100 %): **p <0.01 for significant differences between groups analyzed by the Mann–Whitney post-hoc test

To investigate the potential role that circulating factors related to the SST/CORT system known to be involved in the development of various cancers could play in DMBA-induced tumor development, their plasma levels were evaluated in CORT-KO and SST-KO mice (Fig. 5). It has to be noted that the results shown in this figure represent the average levels of all the mice analyzed in each particular group, including animals with or without tumors, which could present an early or late developmental stage tumor, and could have been sacrificed at different times (according to the tumor size as indicated above). Taking these limitations into consideration, measurements of circulating levels at the time of the sacrifice revealed that plasma levels of GH were significantly elevated in lean CORT-KO mice compared to lean WT mice, whereas in contrast insulin-like growth factor (IGF)-I, prolactin (PRL), insulin and corticosterone levels did not differ significantly among the distinct experimental groups (WT, CORT-KO and SST-KO on a LF or HF diet; Fig. 5).

## Discussion

SST and CORT and their receptors comprise a family of molecules with a clear potential for the identification and development of novel diagnostic/prognostic markers and therapeutic targets, in the treatment and management of several endocrine-related tumors [36–41]. In the case of SST, its synthetic analogs (e.g., octreotide and lanreotide) have been shown to inhibit the proliferation of several tumor-derived cell lines in vitro [5], and to reduce tumor progression in vivo in studies using preclinical models of breast and prostate cancer [42]. Unfortunately, and although SST analogs are widely used for the control of several types of tumors (e.g., pituitary and neuroendocrine tumors [41, 43]), clinical trials in humans with breast tumors using SST analogs have been inconclusive and unsuccessful [8, 44]. On the other hand, although the actions of CORT have commonly been assumed to be similar to those exerted by SST at many levels [4], the potential role of CORT in the control of breast cancer development and progression has not been studied hitherto. Accordingly, this study was designed to investigate the putative contribution of SST and CORT to breast cancer development and progression, especially in the context of an obese phenotype, by using well-characterized mouse models [16, 45, 46]. Taking into account that analysis of spontaneous tumorigenesis in mice would require the use of large numbers of animals and much longer times (due to the low incidence of spontaneous mammary gland tumors in this mouse strain), we used DMBA-treated mouse models, which have been commonly used as models to study mammary tumorigenesis [47]. The results support the notion that endogenous SST and, especially, CORT represent key inhibitory factors in the development and progression of MGT, and their role is markedly influenced by extreme metabolic conditions, such as diet-induced obesity.

Indeed, the current study indicates that CORT would play a more pronounced inhibitory role, compared to SST, in the development of DMBA-induced MG tumors in FVB/N mice under normal (LF diet) conditions, as shown by the different susceptibility of CORT-KO and SST-KO mice in response to tumor induction. Actually, under normal (LF) conditions, SST-KO mice did not differ from WT mice in tumor incidence, latency, multiplicity or burden, despite the fact that some in vitro and in vivo studies have shown that SST analogs can exert inhibitory actions in several cancer types, including pituitary, neuroendocrine and breast tumors [5–7, 48]. In marked contrast, our results demonstrate for the first time that under normal feeding (LF) conditions CORT-KO mice have exacerbated incidence of DMBA-induced MG tumors, thereby demonstrating that CORT and SST exert distinct roles in the control of the tumorigenic process of the MG. Although we cannot exclude the existence of indirect compensatory mechanisms in LF-fed SST-KO mice that could explain the lack of differences in tumor development, these data indicate that CORT may represent a relevant endogenous inhibitor of MGT, acting directly at the MG level or indirectly through changes in other regulatory systems. To identify the causes of these discrepant effects and the mechanisms whereby CORT appears to protect the MG from the development of DMBA-induced tumors, we firstly explored the putative effect of the lack of CORT and SST in the development of the MGs, in terms of complexity and number of TEBs, in that DMBA efficiency has been directly associated with the number of TEBs at the moment of the administration [49]. Our results indicate that under normal conditions young virgin (8-week) and adult (20-week) SST-KO, CORT-KO and control-WT female mice exhibit similar MG complexity and numbers of TEBs, suggesting that MG development is not responsible for the results of DMBA-induced MG tumors observed in the present study. In addition, we have recently reported [24] that under normal feeding conditions, lack of endogenous SST or CORT does not significantly alter local expression of SST/CORT receptors in MGs, or the expression of the main components of the GH/IGF-I system (GH-R, IGF-I, IGF-II, IGF-IR) [24], which play relevant roles in MGT [50] and are regulated by SST/CORT in other tissues [51, 52]. However, at the end of the study (47 weeks of age), SST-KO mice fewer mammary ducts, which could help to explain the scarce incidence of DMBA-induced mammary tumors.

In an attempt to identify circulating factors that could help to explain the exacerbated tumor incidence observed in CORT-KO mice compared to control and SST-KO mice under normal conditions, we determined the plasma levels of a subset of hormones that could be involved in the development and progression of MG tumors, including GH and IGF-I [50], insulin [53], prolactin [54] and corticosterone [55]. Interestingly, CORT-KO mice on a LF diet had clearly elevated circulating GH levels, which may contribute, at least in part, to the increased tumor incidence and multiplicity observed in these mice; however, other additional factors should be also considered. On the other hand, no other comparable, significant changes were observed among groups for the variables examined, perhaps due, at least in part, to the inevitable diversity of the age and condition of the animals at the moment of sacrifice. Actually, the fact that GH levels in SST-KO mice on a LF diet were not augmented as in CORT-KO mice on a LF diet is intriguing, and may relate to a differential response of these mice to DMBA treatment. Nevertheless, this particular divergence in GH levels may help to explain the strikingly distinct responses of CORT-KO and SST-KO mice in tumor incidence, which, in turn, clearly demonstrate that CORT, rather than SST, might be a crucially potent endogenous suppressor of DMBA-induced MGT under normal feeding conditions.

Furthermore, it could be suggested that an SST/CORT compensatory mechanism could take place in CORT-KO and SST-KO models, as our group has previously reported an increase of circulating and stomach levels of endogenous SST in CORT-KO female mice [16]. However, the elevated incidence of mammary tumors in CORT-KO mice would suggest that elevated SST does not seem sufficient to compensate for the lack of endogenous CORT. On the other hand, unfortunately, we cannot confirm the compensatory increase in CORT in SST-KO mice, due to the lack of a reliable means to measure CORT in plasma. Despite this limitation, it seems reasonable that a compensatory increase in CORT levels in SST-KO mice could contribute, at least in part, to the low incidence of mammary tumors in SST-KO mice on a LF diet.

This is not the first report showing distinct actions of CORT and SST [4, 16, 24], which could be likely due to the differential capacity of CORT (compared to SST) to bind and activate separate receptors (such as GHSR, MrgX2 or truncated sst5 variants [56]) and/or even due to additional, as yet unidentified receptors and/or mechanism of CORT action (such as activation of different intracellular signaling pathways, induction of distinctive receptor interactions, etc.). Hence, it is tempting to speculate that the molecular features underlying the divergence of CORT and SST in a functional and pharmacological capacity could be used for the development of novel CORT-like molecules that might be more effective than the actual SST analogs in the treatment of certain endocrine-related cancers, in which CORT could have a predominant inhibitory role.

We also aimed to further explore the putative pathological interaction between the lack of endogenous CORT or SST and MGT in the context of an extreme metabolic condition, such as diet-induced obesity, because the SST/CORT system has been shown to play a crucial role in the regulation of the adaptive mechanism triggered in response to obesity [24, 57], which, in turn, has been directly associated with the development and progression of MG tumorigenic processes in mouse models [58, 59] and in humans [25, 26]. Thus, and in order to validate our experimental model, we first assessed the appropriate status of diet-induced obesity in FVB/N mice, because this mouse strain has been suggested to be less sensitive to the induction of this kind of experimental obesity [60]. However, in this study, HF-fed FVB/N mice had a marked diet-induced obese phenotype, in that mice fed a HF diet had elevated body weight a few weeks after the administration of the HF diet, and these differences remained until the end of the study, compared to genotype-matched LF-fed mice. In support of these results, and reinforcing the idea of the effectiveness of HF diet-induced obesity in the FBV/N mice, plasma levels of leptin (likely, the most relevant adipokine associated with the obese status) were higher in mice on a HF diet compared to those on a LF diet, in line with previous studies reporting strong correlation between leptin levels and adiposity [61]. Moreover, it should be noted that LF and HF diets are micronutrient-matched diets, and therefore, differences would only be attributable to increased dietary fat content and/or subsequent fat storage. Although HF feeding did not induce a significant increment in tumor incidence, multiplicity or burden in WT mice, prevalence of hyperplasic lesions in the MG of mice on the HF diet was significantly higher than that observed in mice on the LF diet, which is consistent with previous studies [62]. Of note, in CORT-KO mice, obese and lean animals exhibited similar susceptibility to DMBA-induced carcinogenesis, suggesting that diet-induced obesity does not exacerbate further the susceptibility generated by lack of CORT, and that maximal levels are already reached under normal feeding conditions.

In clear contrast, lack of SST drastically aggravated tumor incidence in the context of HF diet-induced obesity, in that SST-KO obese mice had marked worsening in DMBA-induced consequences, with higher tumor incidence, multiplicity and hyperplastic lesions than lean SST-KO mice, and with higher tumor incidence than HF diet-fed WT controls. These data suggest that SST can play a pivotal role in the pathological association between obesity-associated changes and MGT.

In line with the above, we also explored the putative contribution of changes in MG development to the differences observed, and found that the HF diet increased the number of TEBs in all the experimental groups (being more pronounced in WT mice), which is consistent with previous studies [33, 63] and could influence the incidence of malignant lesions in the MG in obese mice compared with their lean counterparts. Indeed, MG complexity was especially elevated in SST-KO mice on a HF compared to a LF diet, which might be one of the underlying reasons for the exacerbated incidence of DMBA-induced tumors in this experimental condition.

## Conclusions

Altogether, our data demonstrate that endogenous SST and CORT distinctly contribute to the control of DMBA-induced MG tumorigenesis in mice, and suggest that CORT, rather than SST, might act as a key inhibitory factor of MG tumorigenesis under normal feeding conditions. Our results also suggest that SST, and possibly CORT, might play a pivotal role in the pathological association between obesity-associated changes and MG tumorigenesis. The underlying factors for these different responses of CORT and SST still need to be fully elucidated; however, the data presented herein suggest that CORT-like molecules, rather than SST-like molecules, could be more promising tools for the medical treatment of certain endocrine-related tumors such as breast cancer. Therefore, additional efforts are warranted to identify the specific and distinctive mechanism of action of CORT, as this novel information could pave the way towards the identification and/or development of more useful diagnostic or therapeutic targets in these highly relevant pathological conditions.

## Additional files

Additional file 1: Histopathological analysis of 7, 12 dimethylbenz[α]anthracene (DMBA)-induced mammary gland (MG) tumors. Representative images and histopathological classification of DMBA-induced MG tumors formed in low fat (*LF*)-fed and high-fat (*HF*)-fed wild-type (*WT*), cortistatin (*CORT*)-knockout (*KO*) and somatostatin (*SST*)-KO mice. **a** Adenocarcinoma. **b** Well-differentiated squamous carcinoma. **c** Moderately differentiated squamous carcinoma. **d** undifferentiated carcinoma. *Scale* (*top right*) indicates 100 μm. (PDF 277 kb) Additional file 2: Malignancy features (proliferation rate, inflammation and *de novo* angiogenesis) analysis of 7, 12 dimethylbenz[α]anthracene (DMBA)-induced mammary gland tumors in low fat (*LF*)-fed and high fat (*HF*)-fed wild-type (*WT*), cortistatin (*CORT*)-knockout (*KO*) and somatostatin (*SST*)-KO mice. Values represent mean ± standard error of the mean of percentage of KI67-positive cells (**a**), number of mitotic cells (per each 10 high power fields (HPF)) (**b**), scored inflammatory status evaluating number of inflammatory cells (**C**) and scored *de novo* angiogenesis evaluating number of small blood vessels in a subset of the developed tumors (**d**). Inflammation and *de novo* angiogenesis were valued as 1/3 (scarce), 2/3 (moderate) or 3/3 (abundant). *n.d.* not determined due to the lack of available tumors or the tumoral piece to be evaluated. (PDF 33 kb)

## Abbreviations

ANOVA: analysis of variance
CORT: cortistatin
DMBA: 7, 12 dimethylbenz[α]anthracene
ELISA: enzyme-linked immunosorbent assay
ER: estrogen receptor
GH: growth hormone
HER2: human epidermal growth factor receptor
HF: high fat
IACUC: Institutional Animal Care and Use Committee
IGF-I: insulin-like growth factor 1
KO: knockout
LF: low fat
MG: mammary gland
MGT: mammary gland tumorigenesis
PR: progesterone receptor
PRL: prolactin
SST: somatostatin
TEB: terminal end bud
WT: wild-type
