# Supplementary material for: Lack of cortistatin or somatostatin differentially influences DMBA-induced mammary gland tumorigenesis in mice in an obesity-dependent mode
Source: Breast Cancer Res. 2016 Mar 8;18:29. doi: 10.1186/s13058-016-0689-1 (PMC4782371; doi:10.1186/s13058-016-0689-1)
Supplement: Additional file 1: — Histopathological analysis of 7, 12 dimethylbenz[α]anthracene (DMBA)-induced mammary gland (MG) tumors. Representative images and histopathological classification of DMBA-induced MG tumors formed in low fat (LF)-fed and high-fat (HF)-fed wild-type (WT), cortistatin (CORT)-knockout (KO) and somatostatin (SST)-KO mice. a Adenocarcinoma. b Well-differentiated squamous carcinoma. c Moderately differentiated squamous carcinoma. d undifferentiated carcinoma. Scale (top right) indicates 100 μm. (PDF 277 kb) [file 13058_2016_689_MOESM1_ESM.pdf]

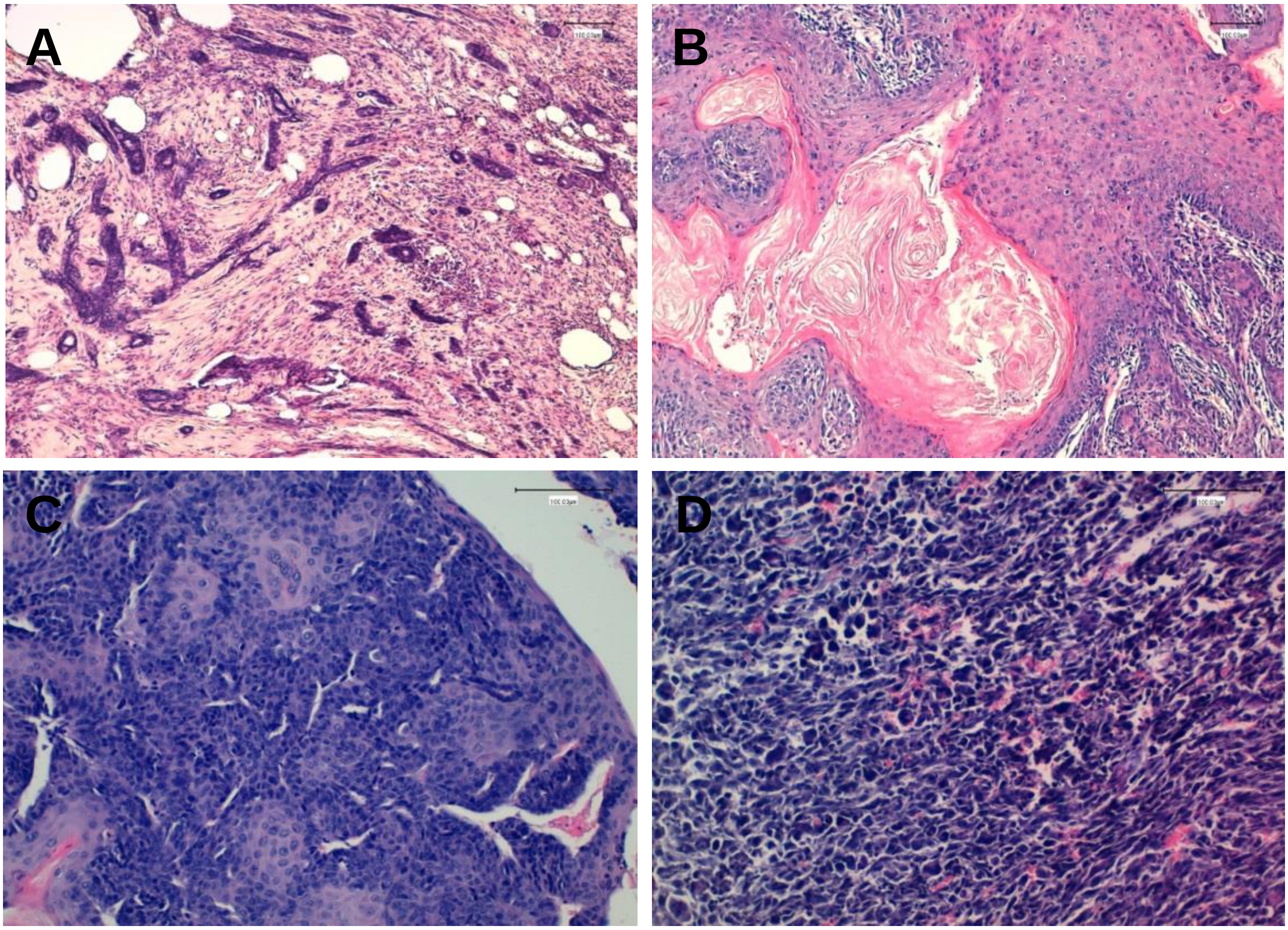

**WT LF (n=3)**	**WT HF (n=2)**	**CORT-KO LF (n=10)**	**CORT-KO HF (n=4)**	**SST-KO LF**	**SST-KO HF (n=4)**
Undifferentiated carcinoma (n=2)	Undifferentiated carcinoma (n=1)	Well-differentiated squamous carcinoma (n=3)	Undifferentiated carcinoma (n=2)	No palpable tumor detected	Undifferentiated carcinoma (n=3)
Well-differentiated squamous carcinoma (n=1)	Moderately-differentiated squamous carcinoma (n=1)	Undifferentiated carcinoma (n=7)	Well-differentiated squamous carcinoma (n=1)		Well-differentiated squamous carcinoma (n=1)
			Moderately-differentiated squamous carcinoma (n=1)		
