# Supplementary figures and images for: Lack of cortistatin or somatostatin differentially influences DMBA-induced mammary gland tumorigenesis in mice in an obesity-dependent mode
Source: Breast Cancer Res. 2016 Mar 8;18:29. doi: 10.1186/s13058-016-0689-1 (PMC4782371; doi:10.1186/s13058-016-0689-1)

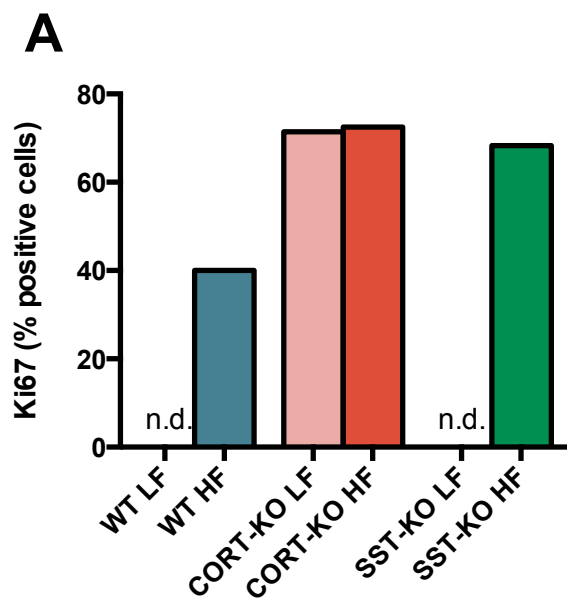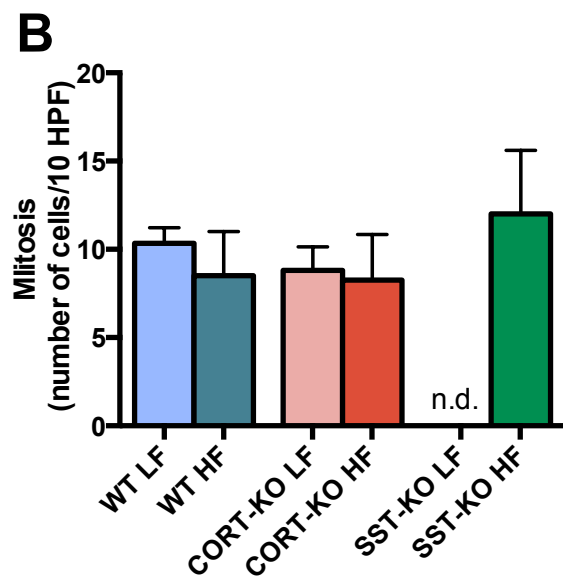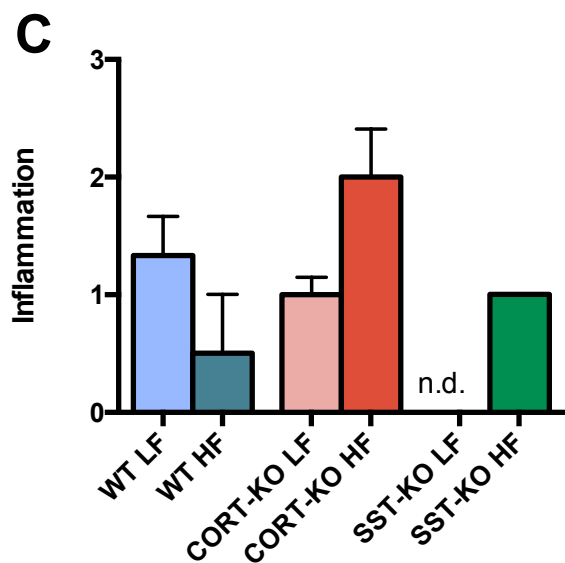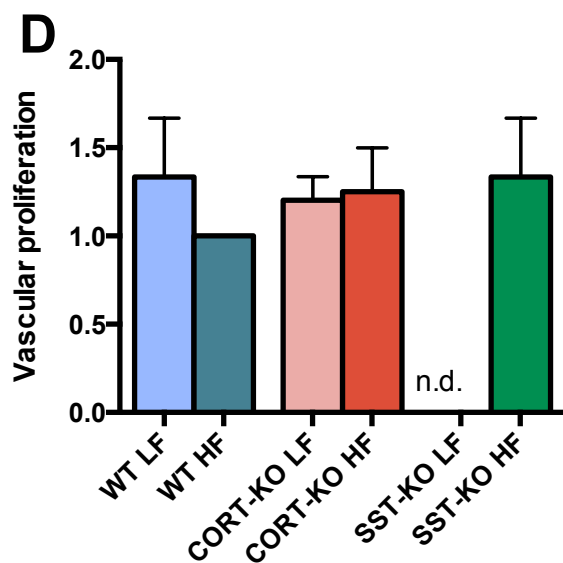

Supplement: Additional file 2: — Malignancy features (proliferation rate, inflammation and de novo angiogenesis) analysis of 7, 12 dimethylbenz[α]anthracene (DMBA)-induced mammary gland tumors in low fat (LF)-fed and high fat (HF)-fed wild-type (WT), cortistatin (CORT)-knockout (KO) and somatostatin (SST)-KO mice. Values represent mean ± standard error of the mean of percentage of KI67-positive cells (a), number of mitotic cells (per each 10 high power fields (HPF)) (b), scored inflammatory status evaluating number of inflammatory cells (C) and scored de novo angiogenesis evaluating number of small blood vessels in a subset of the developed tumors (d). Inflammation and de novo angiogenesis were valued as 1/3 (scarce), 2/3 (moderate) or 3/3 (abundant). n.d. not determined due to the lack of available tumors or the tumoral piece to be evaluated. (PDF 33 kb) [file 13058_2016_689_MOESM2_ESM.pdf]
